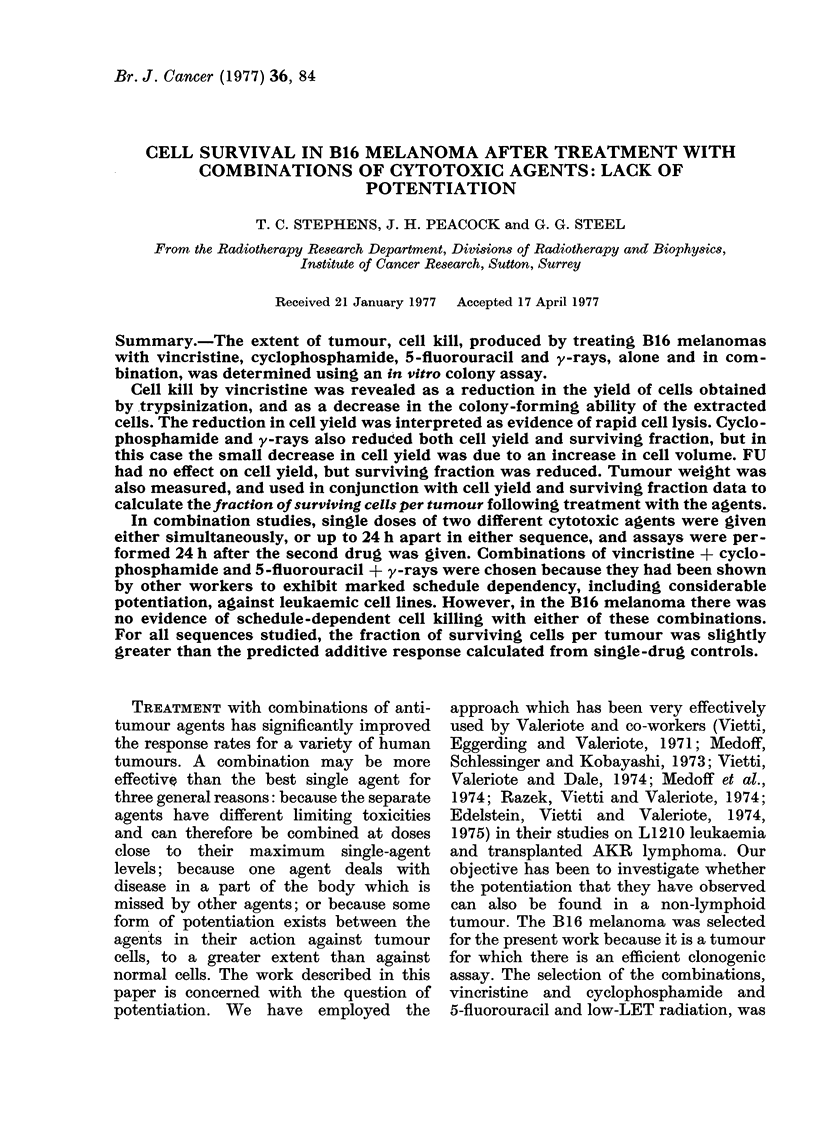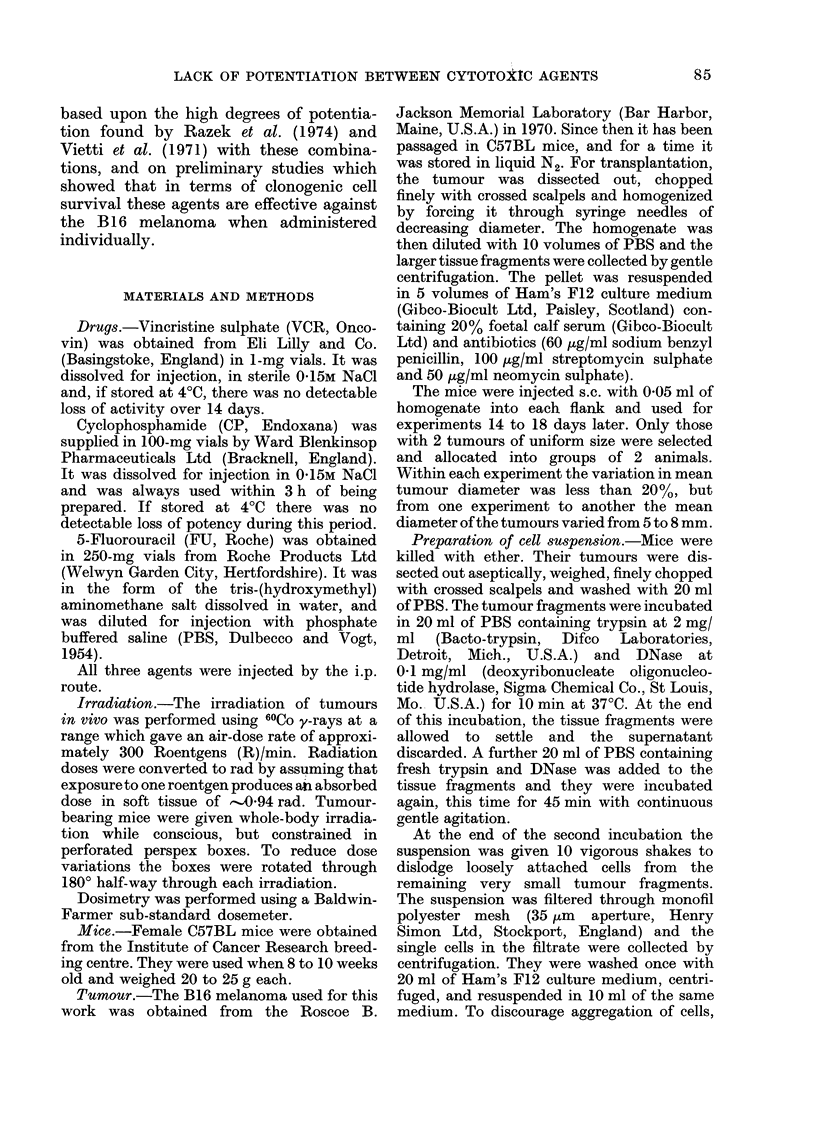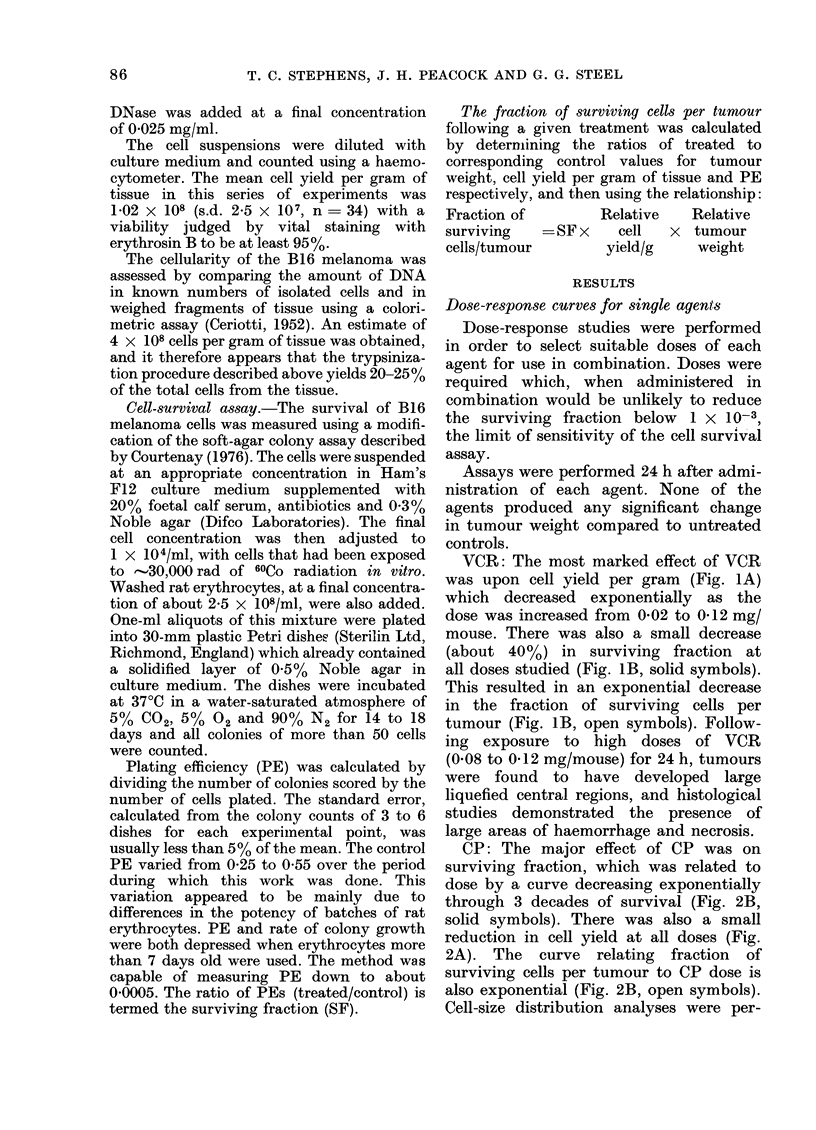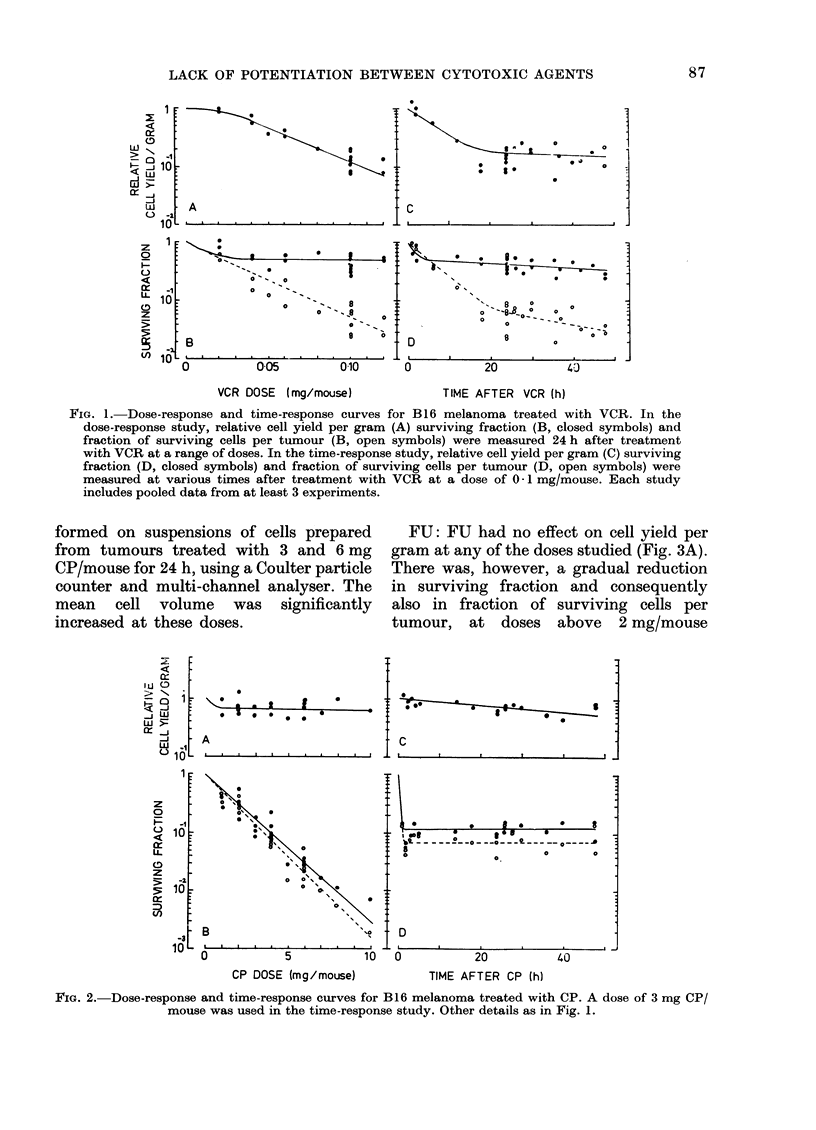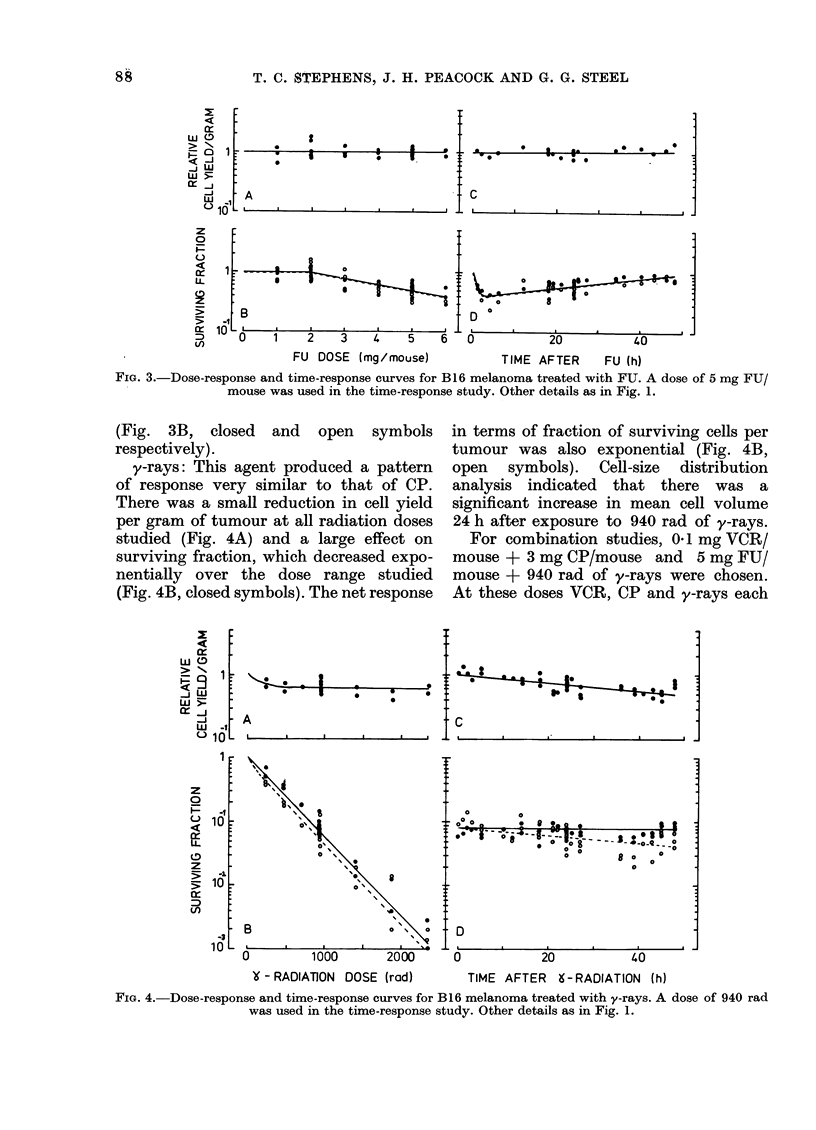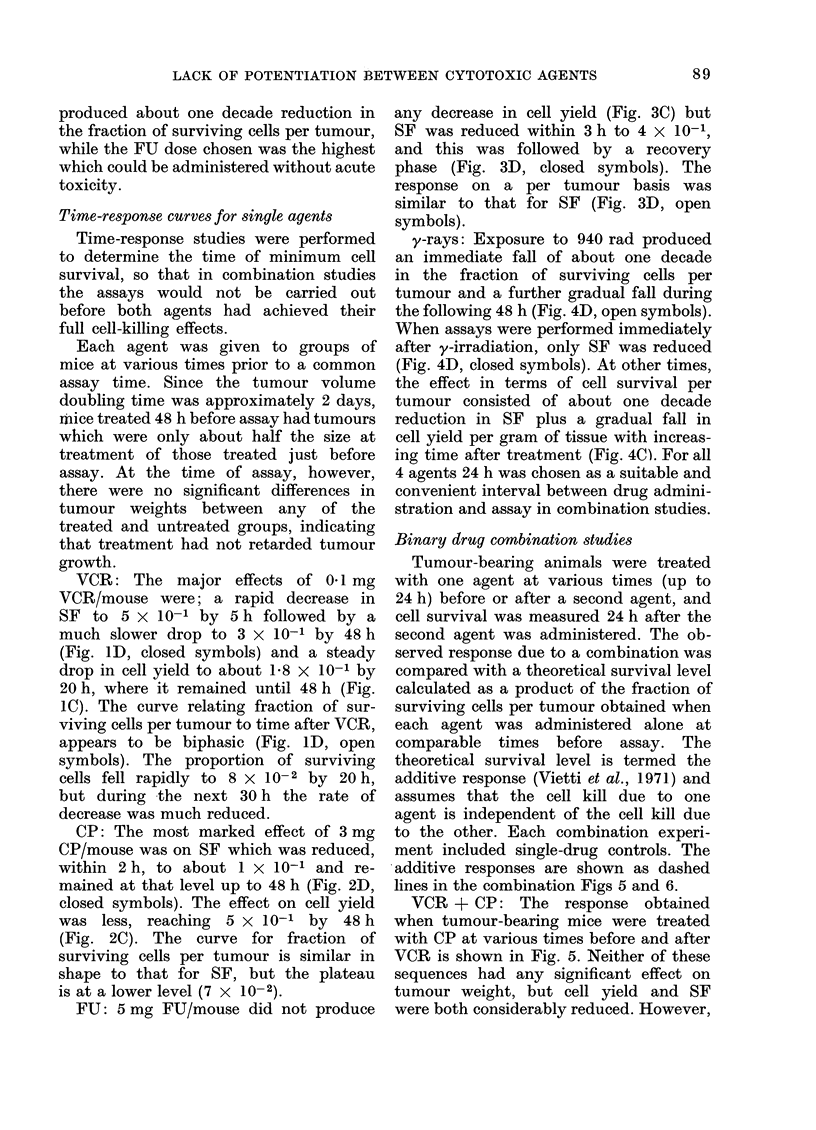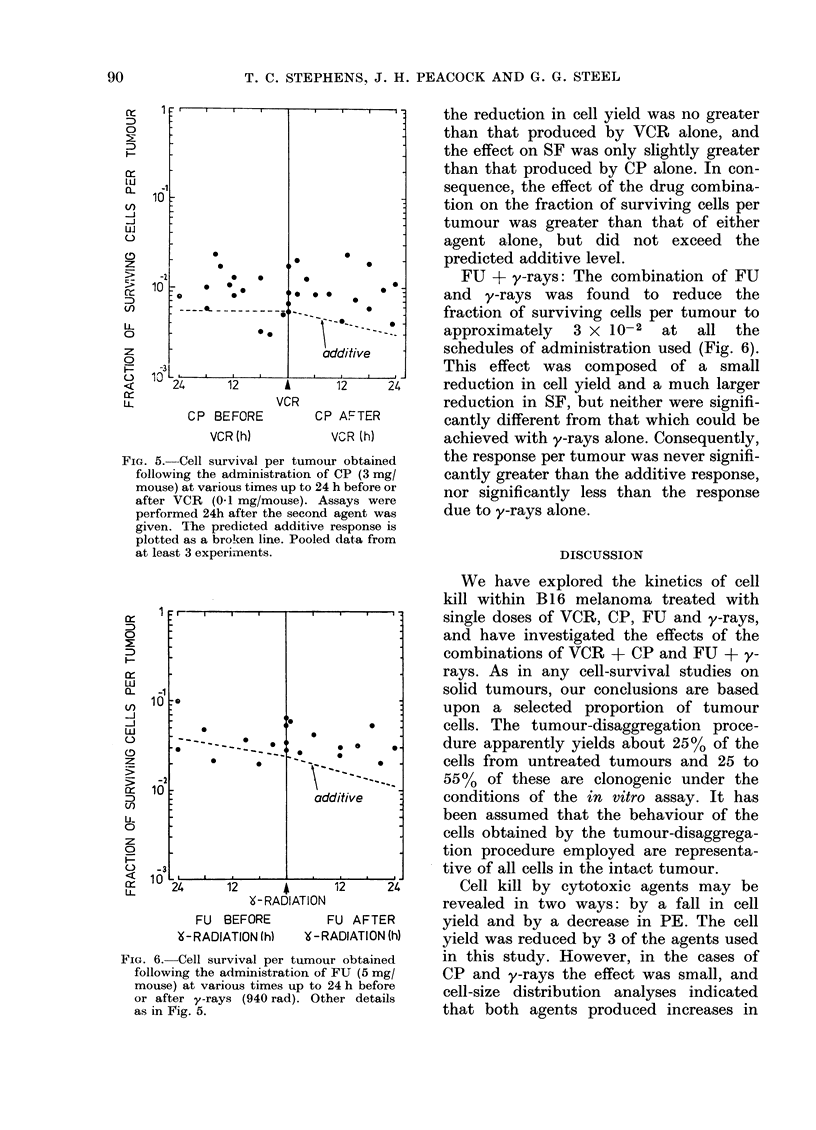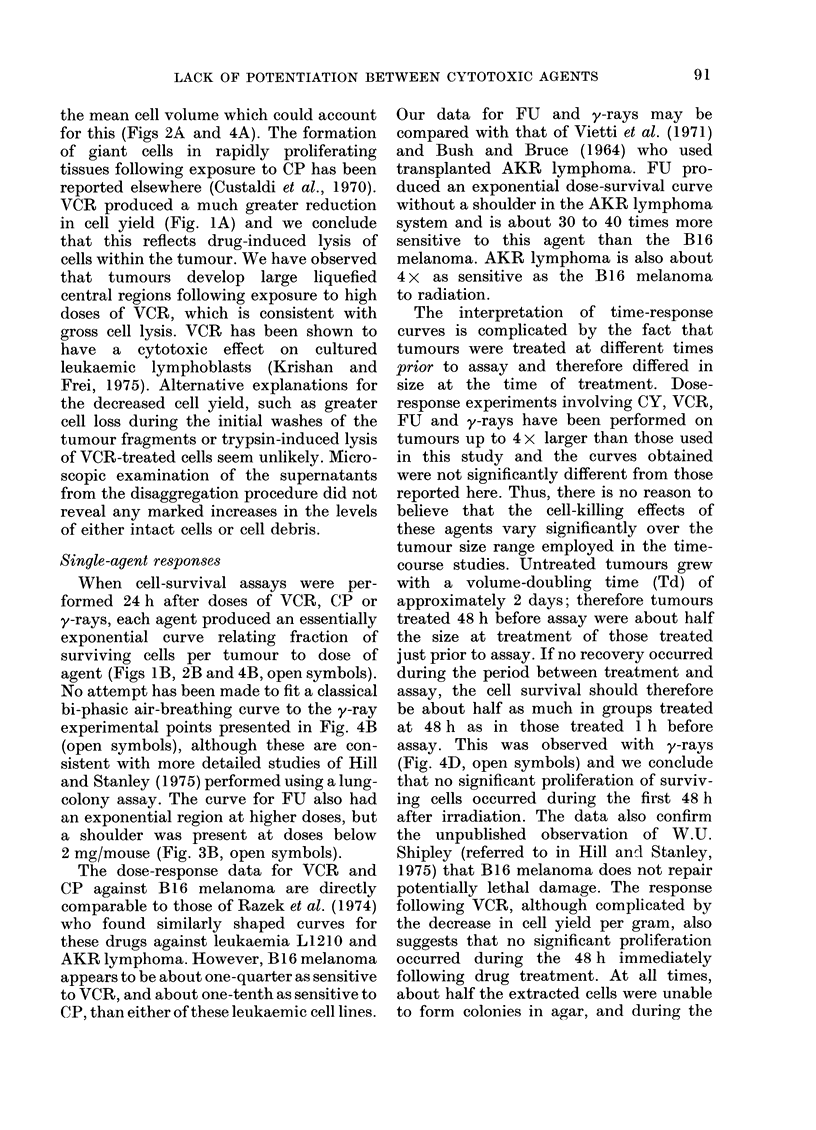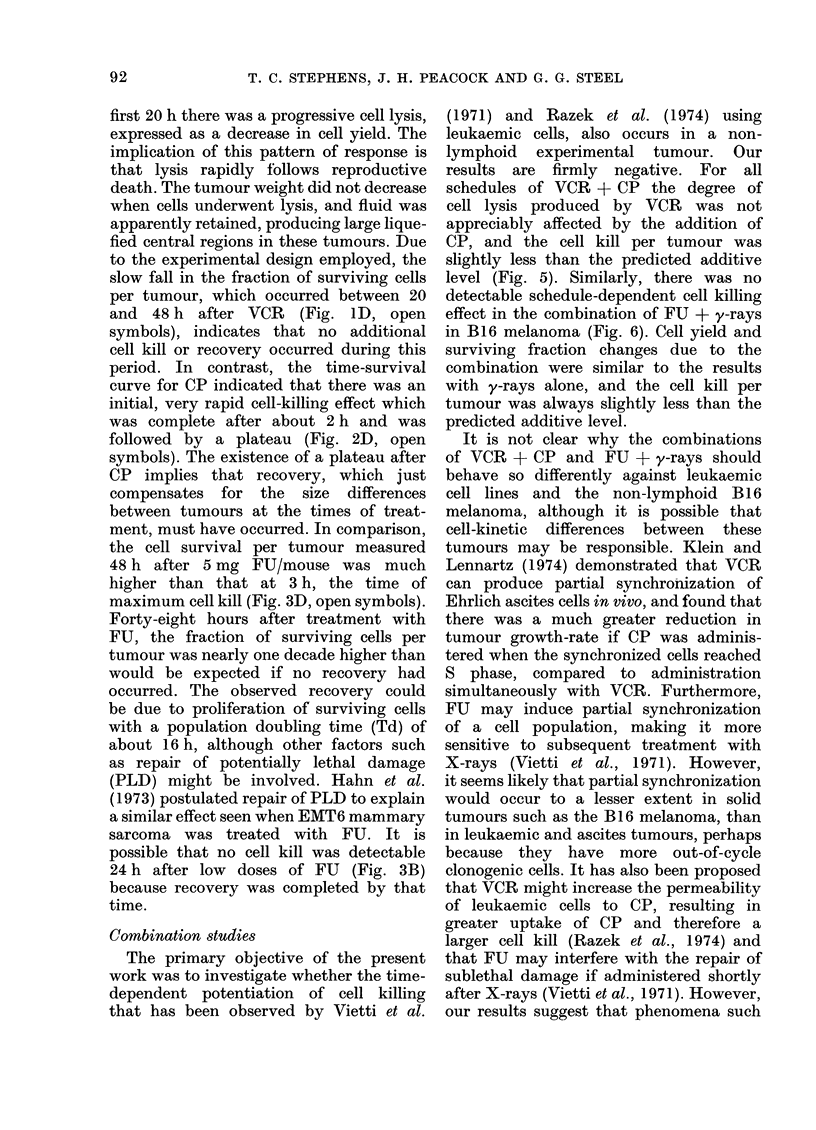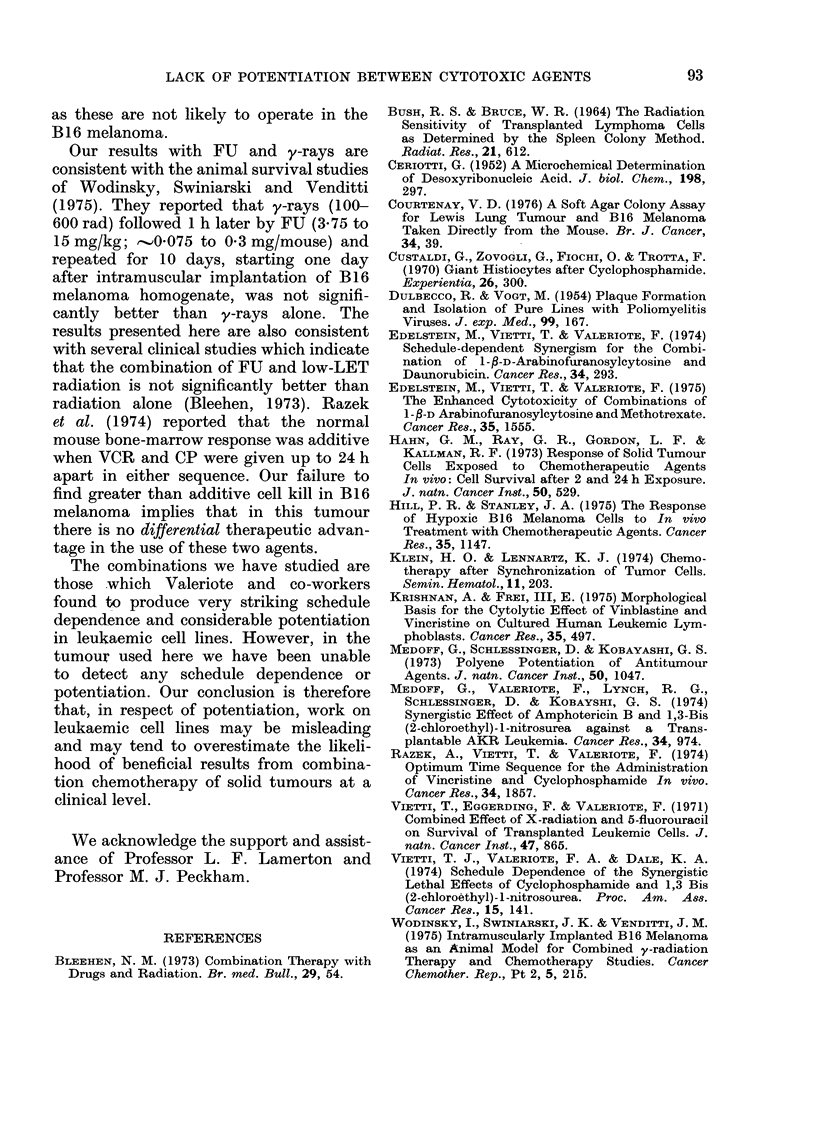# Cell survival in B16 melanoma after treatment with combinations of cytotoxic agents: lack of potentiation.

**DOI:** 10.1038/bjc.1977.158

**Published:** 1977-07

**Authors:** T. C. Stephens, J. H. Peacock, G. G. Steel

## Abstract

The extent of tumour, cell kill, produced by treating B16 melanomas with vincristine, cyclophosphamide, 5-fluorouracil and gamma-rays, alone and in combination, was determined using an in vitro colony assay. Cell kill by vincristine was revealed as a reduction in the yield of cells obtained by trypsinization, and as a decrease in the colony-forming ability of the extracted cells. The reduction in cell yield was interpreted as evidence of rapid cell lysis. Cyclophosphamide and gamma-rays also reduced both cell yield and surviving fraction, but in this case the small decrease in cell yield was due to an increase in cell volume. FU had no effect on cell yield, but surviving fraction was reduced. Tumour weight was also measured, and used in conjunction with cell yield and surviving fraction data to calculate the fraction of surviving cells per tumour following treatment with the agents. In combination studies, single doses of two different cytotoxic agents were given either simultaneously, or up to 24 h apart in either sequence, and assays were performed 24 h after the second drug was given. Combinations of vincristine + cyclophosphamide and 5-fluorouracil + gamma-rays were chosen because they had been shown by other workers to exhibit marked schedule dependency, including considerabl potentiation, against leukaemic cell lines. However, in the B16 melanoma there was no evidence of schedule-dependent cell killing with either of these combinations. For all sequences studied, the fraction of surviving cells per tumour was slightly greater than the predicted additive response calculated from single-drug controls.


*Br. J. Cancer* (1977) **36**, 84

### CELL SURVIVAL IN B16 MELANOMA AFTER TREATMENT WITH COMBINATIONS OF CYTOTOXIC AGENTS: LACK OF POTENTIATION

T. C. STEPHENS, J. H. PEACOCK and G. G. STEEL

*From the Radiotherapy Research Department, Divisions of Radiotherapy and Biophysics, Institute of Cancer Research, Sutton, Surrey*

Received 21 January 1977 Accepted 17 April 1977

**Summary.—The extent of tumour, cell kill, produced by treating B16 melanomas with vincristine, cyclophosphamide, 5-fluorouracil and $\gamma$-rays, alone and in combination, was determined using an *in vitro* colony assay.**

**Cell kill by vincristine was revealed as a reduction in the yield of cells obtained by trypsinization, and as a decrease in the colony-forming ability of the extracted cells. The reduction in cell yield was interpreted as evidence of rapid cell lysis. Cyclophosphamide and $\gamma$-rays also reduced both cell yield and surviving fraction, but in this case the small decrease in cell yield was due to an increase in cell volume. FU had no effect on cell yield, but surviving fraction was reduced. Tumour weight was also measured, and used in conjunction with cell yield and surviving fraction data to calculate the *fraction of surviving cells per tumour* following treatment with the agents.**

**In combination studies, single doses of two different cytotoxic agents were given either simultaneously, or up to 24 h apart in either sequence, and assays were performed 24 h after the second drug was given. Combinations of vincristine + cyclophosphamide and 5-fluorouracil + $\gamma$-rays were chosen because they had been shown by other workers to exhibit marked schedule dependency, including considerable potentiation, against leukaemic cell lines. However, in the B16 melanoma there was no evidence of schedule-dependent cell killing with either of these combinations. For all sequences studied, the fraction of surviving cells per tumour was slightly greater than the predicted additive response calculated from single-drug controls.**

TREATMENT with combinations of anti-tumour agents has significantly improved the response rates for a variety of human tumours. A combination may be more effective than the best single agent for three general reasons: because the separate agents have different limiting toxicities and can therefore be combined at doses close to their maximum single-agent levels; because one agent deals with disease in a part of the body which is missed by other agents; or because some form of potentiation exists between the agents in their action against tumour cells, to a greater extent than against normal cells. The work described in this paper is concerned with the question of potentiation. We have employed the approach which has been very effectively used by Valeriote and co-workers (Vietti, Eggerding and Valeriote, 1971; Medoff, Schlessinger and Kobayashi, 1973; Vietti, Valeriote and Dale, 1974; Medoff *et al.*, 1974; Razek, Vietti and Valeriote, 1974; Edelstein, Vietti and Valeriote, 1974, 1975) in their studies on L1210 leukaemia and transplanted AKR lymphoma. Our objective has been to investigate whether the potentiation that they have observed can also be found in a non-lymphoid tumour. The B16 melanoma was selected for the present work because it is a tumour for which there is an efficient clonogenic assay. The selection of the combinations, vincristine and cyclophosphamide and 5-fluorouracil and low-LET radiation, was based upon the high degrees of potentiation found by Razek *et al.* (1974) and Vietti *et al.* (1971) with these combinations, and on preliminary studies which showed that in terms of clonogenic cell survival these agents are effective against the B16 melanoma when administered individually.

### MATERIALS AND METHODS

*Drugs*.—Vincristine sulphate (VCR, Oncovin) was obtained from Eli Lilly and Co. (Basingstoke, England) in 1-mg vials. It was dissolved for injection, in sterile 0·15M NaCl and, if stored at 4°C, there was no detectable loss of activity over 14 days.

Cyclophosphamide (CP, Endoxana) was supplied in 100-mg vials by Ward Blenkinsop Pharmaceuticals Ltd (Bracknell, England). It was dissolved for injection in 0·15M NaCl and was always used within 3 h of being prepared. If stored at 4°C there was no detectable loss of potency during this period.

5-Fluorouracil (FU, Roche) was obtained in 250-mg vials from Roche Products Ltd (Welwyn Garden City, Hertfordshire). It was in the form of the tris-(hydroxymethyl) aminomethane salt dissolved in water, and was diluted for injection with phosphate buffered saline (PBS, Dulbecco and Vogt, 1954).

All three agents were injected by the i.p. route.

*Irradiation*.—The irradiation of tumours *in vivo* was performed using $^{60}$Co $\gamma$-rays at a range which gave an air-dose rate of approximately 300 Roentgens (R)/min. Radiation doses were converted to rad by assuming that exposure to one roentgen produces an absorbed dose in soft tissue of $\sim$0·94 rad. Tumour-bearing mice were given whole-body irradiation while conscious, but constrained in perforated perspex boxes. To reduce dose variations the boxes were rotated through 180° half-way through each irradiation.

Dosimetry was performed using a Baldwin-Farmer sub-standard dosemeter.

*Mice*.—Female C57BL mice were obtained from the Institute of Cancer Research breeding centre. They were used when 8 to 10 weeks old and weighed 20 to 25 g each.

*Tumour*.—The B16 melanoma used for this work was obtained from the Roscoe B. Jackson Memorial Laboratory (Bar Harbor, Maine, U.S.A.) in 1970. Since then it has been passaged in C57BL mice, and for a time it was stored in liquid $N_2$. For transplantation, the tumour was dissected out, chopped finely with crossed scalpels and homogenized by forcing it through syringe needles of decreasing diameter. The homogenate was then diluted with 10 volumes of PBS and the larger tissue fragments were collected by gentle centrifugation. The pellet was resuspended in 5 volumes of Ham's F12 culture medium (Gibco-Biocult Ltd, Paisley, Scotland) containing 20% foetal calf serum (Gibco-Biocult Ltd) and antibiotics (60 $\mu$g/ml sodium benzyl penicillin, 100 $\mu$g/ml streptomycin sulphate and 50 $\mu$g/ml neomycin sulphate).

The mice were injected s.c. with 0·05 ml of homogenate into each flank and used for experiments 14 to 18 days later. Only those with 2 tumours of uniform size were selected and allocated into groups of 2 animals. Within each experiment the variation in mean tumour diameter was less than 20%, but from one experiment to another the mean diameter of the tumours varied from 5 to 8 mm.

*Preparation of cell suspension*.—Mice were killed with ether. Their tumours were dissected out aseptically, weighed, finely chopped with crossed scalpels and washed with 20 ml of PBS. The tumour fragments were incubated in 20 ml of PBS containing trypsin at 2 mg/ml (Bacto-trypsin, Difco Laboratories, Detroit, Mich., U.S.A.) and DNase at 0·1 mg/ml (deoxyribonucleate oligonucleotide hydrolase, Sigma Chemical Co., St Louis, Mo. U.S.A.) for 10 min at 37°C. At the end of this incubation, the tissue fragments were allowed to settle and the supernatant discarded. A further 20 ml of PBS containing fresh trypsin and DNase was added to the tissue fragments and they were incubated again, this time for 45 min with continuous gentle agitation.

At the end of the second incubation the suspension was given 10 vigorous shakes to dislodge loosely attached cells from the remaining very small tumour fragments. The suspension was filtered through monofil polyester mesh (35 $\mu$m aperture, Henry Simon Ltd, Stockport, England) and the single cells in the filtrate were collected by centrifugation. They were washed once with 20 ml of Ham's F12 culture medium, centrifuged, and resuspended in 10 ml of the same medium. To discourage aggregation of cells,

DNase was added at a final concentration of 0·025 mg/ml.

The cell suspensions were diluted with culture medium and counted using a haemocytometer. The mean cell yield per gram of tissue in this series of experiments was $1{\cdot}02 \times 10^8$ (s.d. $2{\cdot}5 \times 10^7$, n = 34) with a viability judged by vital staining with erythrosin B to be at least 95%.

The cellularity of the B16 melanoma was assessed by comparing the amount of DNA in known numbers of isolated cells and in weighed fragments of tissue using a colorimetric assay (Ceriotti, 1952). An estimate of $4 \times 10^8$ cells per gram of tissue was obtained, and it therefore appears that the trypsinization procedure described above yields 20–25% of the total cells from the tissue.

*Cell-survival assay.*—The survival of B16 melanoma cells was measured using a modification of the soft-agar colony assay described by Courtenay (1976). The cells were suspended at an appropriate concentration in Ham's F12 culture medium supplemented with 20% foetal calf serum, antibiotics and 0·3% Noble agar (Difco Laboratories). The final cell concentration was then adjusted to $1 \times 10^4$/ml, with cells that had been exposed to ~30,000 rad of $^{60}$Co radiation *in vitro.* Washed rat erythrocytes, at a final concentration of about $2{\cdot}5 \times 10^8$/ml, were also added. One-ml aliquots of this mixture were plated into 30-mm plastic Petri dishes (Sterilin Ltd, Richmond, England) which already contained a solidified layer of 0·5% Noble agar in culture medium. The dishes were incubated at 37°C in a water-saturated atmosphere of 5% $CO_2$, 5% $O_2$ and 90% $N_2$ for 14 to 18 days and all colonies of more than 50 cells were counted.

Plating efficiency (PE) was calculated by dividing the number of colonies scored by the number of cells plated. The standard error, calculated from the colony counts of 3 to 6 dishes for each experimental point, was usually less than 5% of the mean. The control PE varied from 0·25 to 0·55 over the period during which this work was done. This variation appeared to be mainly due to differences in the potency of batches of rat erythrocytes. PE and rate of colony growth were both depressed when erythrocytes more than 7 days old were used. The method was capable of measuring PE down to about 0·0005. The ratio of PEs (treated/control) is termed the surviving fraction (SF).

*The fraction of surviving cells per tumour* following a given treatment was calculated by determining the ratios of treated to corresponding control values for tumour weight, cell yield per gram of tissue and PE respectively, and then using the relationship:

$$\text{Fraction of surviving cells/tumour} = \text{SF} \times \text{Relative cell yield/g} \times \text{Relative tumour weight}$$

RESULTS

*Dose-response curves for single agents*

Dose-response studies were performed in order to select suitable doses of each agent for use in combination. Doses were required which, when administered in combination would be unlikely to reduce the surviving fraction below $1 \times 10^{-3}$, the limit of sensitivity of the cell survival assay.

Assays were performed 24 h after administration of each agent. None of the agents produced any significant change in tumour weight compared to untreated controls.

VCR: The most marked effect of VCR was upon cell yield per gram (Fig. 1A) which decreased exponentially as the dose was increased from 0·02 to 0·12 mg/mouse. There was also a small decrease (about 40%) in surviving fraction at all doses studied (Fig. 1B, solid symbols). This resulted in an exponential decrease in the fraction of surviving cells per tumour (Fig. 1B, open symbols). Following exposure to high doses of VCR (0·08 to 0·12 mg/mouse) for 24 h, tumours were found to have developed large liquefied central regions, and histological studies demonstrated the presence of large areas of haemorrhage and necrosis.

CP: The major effect of CP was on surviving fraction, which was related to dose by a curve decreasing exponentially through 3 decades of survival (Fig. 2B, solid symbols). There was also a small reduction in cell yield at all doses (Fig. 2A). The curve relating fraction of surviving cells per tumour to CP dose is also exponential (Fig. 2B, open symbols). Cell-size distribution analyses were per-

FIG. 1.—Dose-response and time-response curves for B16 melanoma treated with VCR. In the dose-response study, relative cell yield per gram (A) surviving fraction (B, closed symbols) and fraction of surviving cells per tumour (B, open symbols) were measured 24 h after treatment with VCR at a range of doses. In the time-response study, relative cell yield per gram (C) surviving fraction (D, closed symbols) and fraction of surviving cells per tumour (D, open symbols) were measured at various times after treatment with VCR at a dose of 0·1 mg/mouse. Each study includes pooled data from at least 3 experiments.

formed on suspensions of cells prepared from tumours treated with 3 and 6 mg CP/mouse for 24 h, using a Coulter particle counter and multi-channel analyser. The mean cell volume was significantly increased at these doses.

FU: FU had no effect on cell yield per gram at any of the doses studied (Fig. 3A). There was, however, a gradual reduction in surviving fraction and consequently also in fraction of surviving cells per tumour, at doses above 2 mg/mouse

FIG. 2.—Dose-response and time-response curves for B16 melanoma treated with CP. A dose of 3 mg CP/ mouse was used in the time-response study. Other details as in Fig. 1.

Fig. 3.—Dose-response and time-response curves for B16 melanoma treated with FU. A dose of 5 mg FU/ mouse was used in the time-response study. Other details as in Fig. 1.

(Fig. 3B, closed and open symbols respectively).

$\gamma$-rays: This agent produced a pattern of response very similar to that of CP. There was a small reduction in cell yield per gram of tumour at all radiation doses studied (Fig. 4A) and a large effect on surviving fraction, which decreased exponentially over the dose range studied (Fig. 4B, closed symbols). The net response in terms of fraction of surviving cells per tumour was also exponential (Fig. 4B, open symbols). Cell-size distribution analysis indicated that there was a significant increase in mean cell volume 24 h after exposure to 940 rad of $\gamma$-rays.

For combination studies, 0·1 mg VCR/ mouse + 3 mg CP/mouse and 5 mg FU/ mouse + 940 rad of $\gamma$-rays were chosen. At these doses VCR, CP and $\gamma$-rays each

Fig. 4.—Dose-response and time-response curves for B16 melanoma treated with $\gamma$-rays. A dose of 940 rad was used in the time-response study. Other details as in Fig. 1.

produced about one decade reduction in the fraction of surviving cells per tumour, while the FU dose chosen was the highest which could be administered without acute toxicity.

*Time-response curves for single agents*

Time-response studies were performed to determine the time of minimum cell survival, so that in combination studies the assays would not be carried out before both agents had achieved their full cell-killing effects.

Each agent was given to groups of mice at various times prior to a common assay time. Since the tumour volume doubling time was approximately 2 days, mice treated 48 h before assay had tumours which were only about half the size at treatment of those treated just before assay. At the time of assay, however, there were no significant differences in tumour weights between any of the treated and untreated groups, indicating that treatment had not retarded tumour growth.

VCR: The major effects of 0·1 mg VCR/mouse were; a rapid decrease in SF to $5 \times 10^{-1}$ by 5 h followed by a much slower drop to $3 \times 10^{-1}$ by 48 h (Fig. 1D, closed symbols) and a steady drop in cell yield to about $1{\cdot}8 \times 10^{-1}$ by 20 h, where it remained until 48 h (Fig. 1C). The curve relating fraction of surviving cells per tumour to time after VCR, appears to be biphasic (Fig. 1D, open symbols). The proportion of surviving cells fell rapidly to $8 \times 10^{-2}$ by 20 h, but during the next 30 h the rate of decrease was much reduced.

CP: The most marked effect of 3 mg CP/mouse was on SF which was reduced, within 2 h, to about $1 \times 10^{-1}$ and remained at that level up to 48 h (Fig. 2D, closed symbols). The effect on cell yield was less, reaching $5 \times 10^{-1}$ by 48 h (Fig. 2C). The curve for fraction of surviving cells per tumour is similar in shape to that for SF, but the plateau is at a lower level ($7 \times 10^{-2}$).

FU: 5 mg FU/mouse did not produce any decrease in cell yield (Fig. 3C) but SF was reduced within 3 h to $4 \times 10^{-1}$, and this was followed by a recovery phase (Fig. 3D, closed symbols). The response on a per tumour basis was similar to that for SF (Fig. 3D, open symbols).

$\gamma$-rays: Exposure to 940 rad produced an immediate fall of about one decade in the fraction of surviving cells per tumour and a further gradual fall during the following 48 h (Fig. 4D, open symbols). When assays were performed immediately after $\gamma$-irradiation, only SF was reduced (Fig. 4D, closed symbols). At other times, the effect in terms of cell survival per tumour consisted of about one decade reduction in SF plus a gradual fall in cell yield per gram of tissue with increasing time after treatment (Fig. 4C). For all 4 agents 24 h was chosen as a suitable and convenient interval between drug administration and assay in combination studies.

*Binary drug combination studies*

Tumour-bearing animals were treated with one agent at various times (up to 24 h) before or after a second agent, and cell survival was measured 24 h after the second agent was administered. The observed response due to a combination was compared with a theoretical survival level calculated as a product of the fraction of surviving cells per tumour obtained when each agent was administered alone at comparable times before assay. The theoretical survival level is termed the additive response (Vietti *et al.*, 1971) and assumes that the cell kill due to one agent is independent of the cell kill due to the other. Each combination experiment included single-drug controls. The additive responses are shown as dashed lines in the combination Figs 5 and 6.

VCR + CP: The response obtained when tumour-bearing mice were treated with CP at various times before and after VCR is shown in Fig. 5. Neither of these sequences had any significant effect on tumour weight, but cell yield and SF were both considerably reduced. However,

FIG. 5.—Cell survival per tumour obtained following the administration of CP (3 mg/mouse) at various times up to 24 h before or after VCR (0·1 mg/mouse). Assays were performed 24h after the second agent was given. The predicted additive response is plotted as a broken line. Pooled data from at least 3 experiments.

FIG. 6.—Cell survival per tumour obtained following the administration of FU (5 mg/mouse) at various times up to 24 h before or after $\gamma$-rays (940 rad). Other details as in Fig. 5.

the reduction in cell yield was no greater than that produced by VCR alone, and the effect on SF was only slightly greater than that produced by CP alone. In consequence, the effect of the drug combination on the fraction of surviving cells per tumour was greater than that of either agent alone, but did not exceed the predicted additive level.

FU + $\gamma$-rays: The combination of FU and $\gamma$-rays was found to reduce the fraction of surviving cells per tumour to approximately $3 \times 10^{-2}$ at all the schedules of administration used (Fig. 6). This effect was composed of a small reduction in cell yield and a much larger reduction in SF, but neither were significantly different from that which could be achieved with $\gamma$-rays alone. Consequently, the response per tumour was never significantly greater than the additive response, nor significantly less than the response due to $\gamma$-rays alone.

### DISCUSSION

We have explored the kinetics of cell kill within B16 melanoma treated with single doses of VCR, CP, FU and $\gamma$-rays, and have investigated the effects of the combinations of VCR + CP and FU + $\gamma$-rays. As in any cell-survival studies on solid tumours, our conclusions are based upon a selected proportion of tumour cells. The tumour-disaggregation procedure apparently yields about 25% of the cells from untreated tumours and 25 to 55% of these are clonogenic under the conditions of the *in vitro* assay. It has been assumed that the behaviour of the cells obtained by the tumour-disaggregation procedure employed are representative of all cells in the intact tumour.

Cell kill by cytotoxic agents may be revealed in two ways: by a fall in cell yield and by a decrease in PE. The cell yield was reduced by 3 of the agents used in this study. However, in the cases of CP and $\gamma$-rays the effect was small, and cell-size distribution analyses indicated that both agents produced increases in the mean cell volume which could account for this (Figs 2A and 4A). The formation of giant cells in rapidly proliferating tissues following exposure to CP has been reported elsewhere (Custaldi *et al.*, 1970). VCR produced a much greater reduction in cell yield (Fig. 1A) and we conclude that this reflects drug-induced lysis of cells within the tumour. We have observed that tumours develop large liquefied central regions following exposure to high doses of VCR, which is consistent with gross cell lysis. VCR has been shown to have a cytotoxic effect on cultured leukaemic lymphoblasts (Krishan and Frei, 1975). Alternative explanations for the decreased cell yield, such as greater cell loss during the initial washes of the tumour fragments or trypsin-induced lysis of VCR-treated cells seem unlikely. Microscopic examination of the supernatants from the disaggregation procedure did not reveal any marked increases in the levels of either intact cells or cell debris.

*Single-agent responses*

When cell-survival assays were performed 24 h after doses of VCR, CP or $\gamma$-rays, each agent produced an essentially exponential curve relating fraction of surviving cells per tumour to dose of agent (Figs 1B, 2B and 4B, open symbols). No attempt has been made to fit a classical bi-phasic air-breathing curve to the $\gamma$-ray experimental points presented in Fig. 4B (open symbols), although these are consistent with more detailed studies of Hill and Stanley (1975) performed using a lung-colony assay. The curve for FU also had an exponential region at higher doses, but a shoulder was present at doses below 2 mg/mouse (Fig. 3B, open symbols).

The dose-response data for VCR and CP against B16 melanoma are directly comparable to those of Razek *et al.* (1974) who found similarly shaped curves for these drugs against leukaemia L1210 and AKR lymphoma. However, B16 melanoma appears to be about one-quarter as sensitive to VCR, and about one-tenth as sensitive to CP, than either of these leukaemic cell lines. Our data for FU and $\gamma$-rays may be compared with that of Vietti *et al.* (1971) and Bush and Bruce (1964) who used transplanted AKR lymphoma. FU produced an exponential dose-survival curve without a shoulder in the AKR lymphoma system and is about 30 to 40 times more sensitive to this agent than the B16 melanoma. AKR lymphoma is also about $4\times$ as sensitive as the B16 melanoma to radiation.

The interpretation of time-response curves is complicated by the fact that tumours were treated at different times *prior* to assay and therefore differed in size at the time of treatment. Dose-response experiments involving CY, VCR, FU and $\gamma$-rays have been performed on tumours up to $4\times$ larger than those used in this study and the curves obtained were not significantly different from those reported here. Thus, there is no reason to believe that the cell-killing effects of these agents vary significantly over the tumour size range employed in the time-course studies. Untreated tumours grew with a volume-doubling time (Td) of approximately 2 days; therefore tumours treated 48 h before assay were about half the size at treatment of those treated just prior to assay. If no recovery occurred during the period between treatment and assay, the cell survival should therefore be about half as much in groups treated at 48 h as in those treated 1 h before assay. This was observed with $\gamma$-rays (Fig. 4D, open symbols) and we conclude that no significant proliferation of surviving cells occurred during the first 48 h after irradiation. The data also confirm the unpublished observation of W.U. Shipley (referred to in Hill and Stanley, 1975) that B16 melanoma does not repair potentially lethal damage. The response following VCR, although complicated by the decrease in cell yield per gram, also suggests that no significant proliferation occurred during the 48 h immediately following drug treatment. At all times, about half the extracted cells were unable to form colonies in agar, and during the first 20 h there was a progressive cell lysis, expressed as a decrease in cell yield. The implication of this pattern of response is that lysis rapidly follows reproductive death. The tumour weight did not decrease when cells underwent lysis, and fluid was apparently retained, producing large liquefied central regions in these tumours. Due to the experimental design employed, the slow fall in the fraction of surviving cells per tumour, which occurred between 20 and 48 h after VCR (Fig. 1D, open symbols), indicates that no additional cell kill or recovery occurred during this period. In contrast, the time-survival curve for CP indicated that there was an initial, very rapid cell-killing effect which was complete after about 2 h and was followed by a plateau (Fig. 2D, open symbols). The existence of a plateau after CP implies that recovery, which just compensates for the size differences between tumours at the times of treatment, must have occurred. In comparison, the cell survival per tumour measured 48 h after 5 mg FU/mouse was much higher than that at 3 h, the time of maximum cell kill (Fig. 3D, open symbols). Forty-eight hours after treatment with FU, the fraction of surviving cells per tumour was nearly one decade higher than would be expected if no recovery had occurred. The observed recovery could be due to proliferation of surviving cells with a population doubling time (Td) of about 16 h, although other factors such as repair of potentially lethal damage (PLD) might be involved. Hahn *et al.* (1973) postulated repair of PLD to explain a similar effect seen when EMT6 mammary sarcoma was treated with FU. It is possible that no cell kill was detectable 24 h after low doses of FU (Fig. 3B) because recovery was completed by that time.

*Combination studies*

The primary objective of the present work was to investigate whether the time-dependent potentiation of cell killing that has been observed by Vietti *et al.* (1971) and Razek *et al.* (1974) using leukaemic cells, also occurs in a non-lymphoid experimental tumour. Our results are firmly negative. For all schedules of VCR + CP the degree of cell lysis produced by VCR was not appreciably affected by the addition of CP, and the cell kill per tumour was slightly less than the predicted additive level (Fig. 5). Similarly, there was no detectable schedule-dependent cell killing effect in the combination of FU + $\gamma$-rays in B16 melanoma (Fig. 6). Cell yield and surviving fraction changes due to the combination were similar to the results with $\gamma$-rays alone, and the cell kill per tumour was always slightly less than the predicted additive level.

It is not clear why the combinations of VCR + CP and FU + $\gamma$-rays should behave so differently against leukaemic cell lines and the non-lymphoid B16 melanoma, although it is possible that cell-kinetic differences between these tumours may be responsible. Klein and Lennartz (1974) demonstrated that VCR can produce partial synchronization of Ehrlich ascites cells *in vivo*, and found that there was a much greater reduction in tumour growth-rate if CP was administered when the synchronized cells reached S phase, compared to administration simultaneously with VCR. Furthermore, FU may induce partial synchronization of a cell population, making it more sensitive to subsequent treatment with X-rays (Vietti *et al.*, 1971). However, it seems likely that partial synchronization would occur to a lesser extent in solid tumours such as the B16 melanoma, than in leukaemic and ascites tumours, perhaps because they have more out-of-cycle clonogenic cells. It has also been proposed that VCR might increase the permeability of leukaemic cells to CP, resulting in greater uptake of CP and therefore a larger cell kill (Razek *et al.*, 1974) and that FU may interfere with the repair of sublethal damage if administered shortly after X-rays (Vietti *et al.*, 1971). However, our results suggest that phenomena such as these are not likely to operate in the B16 melanoma.

Our results with FU and $\gamma$-rays are consistent with the animal survival studies of Wodinsky, Swiniarski and Venditti (1975). They reported that $\gamma$-rays (100–600 rad) followed 1 h later by FU (3·75 to 15 mg/kg; ~0·075 to 0·3 mg/mouse) and repeated for 10 days, starting one day after intramuscular implantation of B16 melanoma homogenate, was not significantly better than $\gamma$-rays alone. The results presented here are also consistent with several clinical studies which indicate that the combination of FU and low-LET radiation is not significantly better than radiation alone (Bleehen, 1973). Razek *et al.* (1974) reported that the normal mouse bone-marrow response was additive when VCR and CP were given up to 24 h apart in either sequence. Our failure to find greater than additive cell kill in B16 melanoma implies that in this tumour there is no *differential* therapeutic advantage in the use of these two agents.

The combinations we have studied are those which Valeriote and co-workers found to produce very striking schedule dependence and considerable potentiation in leukaemic cell lines. However, in the tumour used here we have been unable to detect any schedule dependence or potentiation. Our conclusion is therefore that, in respect of potentiation, work on leukaemic cell lines may be misleading and may tend to overestimate the likelihood of beneficial results from combination chemotherapy of solid tumours at a clinical level.

We acknowledge the support and assistance of Professor L. F. Lamerton and Professor M. J. Peckham.

### REFERENCES

BLEEHEN, N. M. (1973) Combination Therapy with Drugs and Radiation. *Br. med. Bull.*, **29**, 54.

BUSH, R. S. & BRUCE, W. R. (1964) The Radiation Sensitivity of Transplanted Lymphoma Cells as Determined by the Spleen Colony Method. *Radiat. Res.*, **21**, 612.

CERIOTTI, G. (1952) A Microchemical Determination of Desoxyribonucleic Acid. *J. biol. Chem.*, **198**, 297.

COURTENAY, V. D. (1976) A Soft Agar Colony Assay for Lewis Lung Tumour and B16 Melanoma Taken Directly from the Mouse. *Br. J. Cancer*, **34**, 39.

CUSTALDI, G., ZOVOGLI, G., FIOCHI, O. & TROTTA, F. (1970) Giant Histiocytes after Cyclophosphamide. *Experientia*, **26**, 300.

DULBECCO, R. & VOGT, M. (1954) Plaque Formation and Isolation of Pure Lines with Poliomyelitis Viruses. *J. exp. Med.*, **99**, 167.

EDELSTEIN, M., VIETTI, T. & VALERIOTE, F. (1974) Schedule-dependent Synergism for the Combination of 1-$\beta$-D-Arabinofuranosylcytosine and Daunorubicin. *Cancer Res.*, **34**, 293.

EDELSTEIN, M., VIETTI, T. & VALERIOTE, F. (1975) The Enhanced Cytotoxicity of Combinations of 1-$\beta$-D Arabinofuranosylcytosine and Methotrexate. *Cancer Res.*, **35**, 1555.

HAHN, G. M., RAY, G. R., GORDON, L. F. & KALLMAN, R. F. (1973) Response of Solid Tumour Cells Exposed to Chemotherapeutic Agents *In vivo*: Cell Survival after 2 and 24 h Exposure. *J. natn. Cancer Inst.*, **50**, 529.

HILL, P. R. & STANLEY, J. A. (1975) The Response of Hypoxic B16 Melanoma Cells to *In vivo* Treatment with Chemotherapeutic Agents. *Cancer Res.*, **35**, 1147.

KLEIN, H. O. & LENNARTZ, K. J. (1974) Chemotherapy after Synchronization of Tumor Cells. *Semin. Hematol.*, **11**, 203.

KRISHNAN, A. & FREI, III, E. (1975) Morphological Basis for the Cytolytic Effect of Vinblastine and Vincristine on Cultured Human Leukemic Lymphoblasts. *Cancer Res.*, **35**, 497.

MEDOFF, G., SCHLESSINGER, D. & KOBAYASHI, G. S. (1973) Polyene Potentiation of Antitumour Agents. *J. natn. Cancer Inst.*, **50**, 1047.

MEDOFF, G., VALERIOTE, F., LYNCH, R. G., SCHLESSINGER, D. & KOBAYSHI, G. S. (1974) Synergistic Effect of Amphotericin B and 1,3-Bis (2-chloroethyl)-1-nitrosurea against a Transplantable AKR Leukemia. *Cancer Res.*, **34**, 974.

RAZEK, A., VIETTI, T. & VALERIOTE, F. (1974) Optimum Time Sequence for the Administration of Vincristine and Cyclophosphamide *In vivo*. *Cancer Res.*, **34**, 1857.

VIETTI, T., EGGERDING, F. & VALERIOTE, F. (1971) Combined Effect of X-radiation and 5-fluorouracil on Survival of Transplanted Leukemic Cells. *J. natn. Cancer Inst.*, **47**, 865.

VIETTI, T. J., VALERIOTE, F. A. & DALE, K. A. (1974) Schedule Dependence of the Synergistic Lethal Effects of Cyclophosphamide and 1,3 Bis (2-chloroëthyl)-1-nitrosourea. *Proc. Am. Ass. Cancer Res.*, **15**, 141.

WODINSKY, I., SWINIARSKI, J. K. & VENDITTI, J. M. (1975) Intramuscularly Implanted B16 Melanoma as an Animal Model for Combined $\gamma$-radiation Therapy and Chemotherapy Studies. *Cancer Chemother. Rep.*, Pt 2, **5**, 215.